# Why is That Chin Numb? A Case of Burkitt’s Lymphoma Presenting as Numb Chin Syndrome

**DOI:** 10.7759/cureus.10243

**Published:** 2020-09-04

**Authors:** Sejal Neel, Abhi Chand Lohana, Zainab Abbasi, Marcos Madeiro

**Affiliations:** 1 Medicine, Liaquat University of Medical and Health Sciences, Jamshoro, PAK; 2 Surgery, Jinnah Sindh Medical University, Karachi, PAK; 3 Internal Medicine, Liaquat University of Medical and Health Sciences, Jamshoro, PAK; 4 Internal Medicine, Bronson Methodist Hospital, Kalamazoo, USA

**Keywords:** numb chin, burkitt's lymphoma

## Abstract

We report a case of numb chin syndrome (NCS) that preceded the diagnosis of Burkitt’s lymphoma (BL) and discuss our findings with emphasis on metastatic malignancies in general and BL in particular causing NCS. A 73-year-old woman presented with worsening right-sided back pain for a week along with right-sided leg weakness and a progressive thigh and perianal numbness. She reported numbness of the chin which started two weeks prior. MRI showed compression of the cauda equina which was highly suggestive of neoplastic process. After debulking mass surgery, biopsy of the tissue from spine revealed BL. The patient received a round of radiotherapy of spine and multiple rounds of chemotherapy. Six months later she had reoccurrence of lymphoma with metastasis and eventually passed away. Presence of NCS in the setting of an underlying malignancy indicates very poor prognosis. In appropriate clinical setting, NCS should trigger work up for an underlying malignancy.

## Introduction

The numb chin syndrome (NCS) is a rare neurological condition characterized by localized numbness and prickling sensation in the skin of the chin and lower lip, limited to the region served by the mental nerve [1]. The first case of this syndrome was introduced in 1830 by Charles Bell [2]. NCS is mostly caused by infiltration or compression of mental nerve by dental disease but the rare and more important causes include the metastasis of distant tumor such as breast cancer and lymphomas. This syndrome may be the first symptom of underlying malignancy or the first sign of recurrence and metastasis in patients with pre-existing cancer. NCS has also been associated with other systematic diseases such as diabetes and multiple sclerosis. It usually presents unilaterally, but bilateral manifestations have also been reported [1].

Burkitt’s lymphoma (BL) is a type of B-cell non-Hodgkin lymphoma that most commonly arise from chromosomal translocation of C-myc gene from chromosome 14 to chromosome 8 leading to over expression of MYC protein and increased proliferation of B-cells [3]. Some 1%-5% of all non-Hodgkin lymphomas are BL. Patients clinically present with bulky extra nodal disease, bone marrow infiltration, and central nervous system involvement [4].

## Case presentation

A 73-year-old woman with past medical history of hypertension, hyperlipidemia, and hypothyroidism presented with chief complaint of worsening low right-sided back pain for one week. She had not had any trauma or injury to the area prior to onset of pain. She then developed right-sided leg weakness, right thigh numbness, and perianal numbness in a progressive fashion. She also reported urinary retention.

On further questioning, she reported numbness of chin on both sides which started two weeks prior. She did have a left molar tooth extraction one week prior to the onset of all symptoms, and did receive local anesthesia for that. However, she confirmed that the numbness of chin was present prior to tooth extraction as well.

Her vital signs at the time of presentation were: blood pressure 124/58 mmHg, heart rate 89 beats/min, temperature 98.3°F, and respiratory rate 22 breaths/min with oxygen saturation of 95% on room air. Her physical exam was significant for tenderness to palpation of lower lumbar spine, decreased motor strength, and sensation of the right lower extremity. She also had decreased sensation to touch and pain around her lips and on chin bilaterally.

Laboratory investigations at the time of presentation and on Day 4 and Day 5 are listed in Table 1.

**Table 1:** Blood laboratory investigation on day of admission (Day 0), Day 4, and Day 5.

Test name	Day 0	Day 4	Day 5	Reference range
Serum sodium	128 (L)	140	140	135-145 mmol/L
Serum potassium	4.7	4.3	4.5	3.5-5.3 mmol/L
Serum chloride	90 (L)	107	107	98-108 mmol/L
Serum bicarbonate	20 (L)	19 (L)	17 (L)	23-32 mmol/L
Serum creatinine	1.39 (H)	1.24 (H)	0.97	0.60-1.10 mg/dL
Estimated glomerular filtration rate	37 (L)	42 (L)	56 (L)	>60 mL/min
Serum blood urea nitrogen	17	36 (H)	24 (H)	8-23 mg/dL
Serum calcium	10.6 (H)	8.4 (L)	8.9	8.6-10.3 mg/dL
Serum phosphorus		3.7	2.6 (L)	2.7-4.5 mg/dL
Serum magnesium		2.0	1.6 (L)	1.7-2.8 mg/dL
Serum alkaline phosphatase		59	64	39-190 U/L
Serum albumin		2.8 (L)	3.0 (L)	3.5-5.0 g/dL
Serum uric acid		21.2 (H)	10.9 (H)	2.5-8.0 mg/dL
Serum aspartate aminotransferase		70 (H)	88 (H)	0-37 U/L
Serum alanine aminotransferase		23	32	6-37 U/L
Serum lactate dehydrogenase		1,906 (H)	2,114 (H)	94-250 U/L
C-Reactive protein		101.4 (H)		<6.0 mg/L
Serum lactic acid	2.4			0.7-2.5 mmol/L
White blood cell count	6.9	6.6	7.6	4.0-11.0 10*9/L
Red blood cell count	4.41	3.02 (L)	3.27 (L)	4.2-5.5 10*12/L
Hemoglobin	13.0	9.1 (L)	9.7 (L)	12.0-16.0 g/dL
Platelet count	157	128 (L)	138 (L)	140-440 10*9/L
Haptoglobin		238 (H)		30-200 mg/dL
Erythrocyte sedimentation rate	22	39 (H)		<30 mm/h
D-Dimer		1.21 (H)		<0.5 ug/mL [FEU]
Fibrinogen		425		200-450 mg/dL
Schistocytes		None seen		None seen /[HPF]
Prothrombin time	11.7	10.9		10.5-13.0 s
International normalized ratio	1.0	0.9		N/A
Hepatitis A IgM		Negative		Negative
Hepatitis B surface antigen		Negative		Negative
Hepatitis B surface antibody		<3.5 (L)		>11.4 m[iU]/mL
Hepatitis B core antibody		Negative		Negative
Hepatitis B core IgM		Negative		Negative
Hepatitis C antibody		Negative		Negative
HIV-1/HIV-2 antibody		Negative		Negative

She had a CT scan of lumbar spine without contrast which showed multilevel chronic changes without any acute pathology. She underwent a CT myelogram of the lumbar spine, which also did not show any acute pathology.

**Figure 1:**
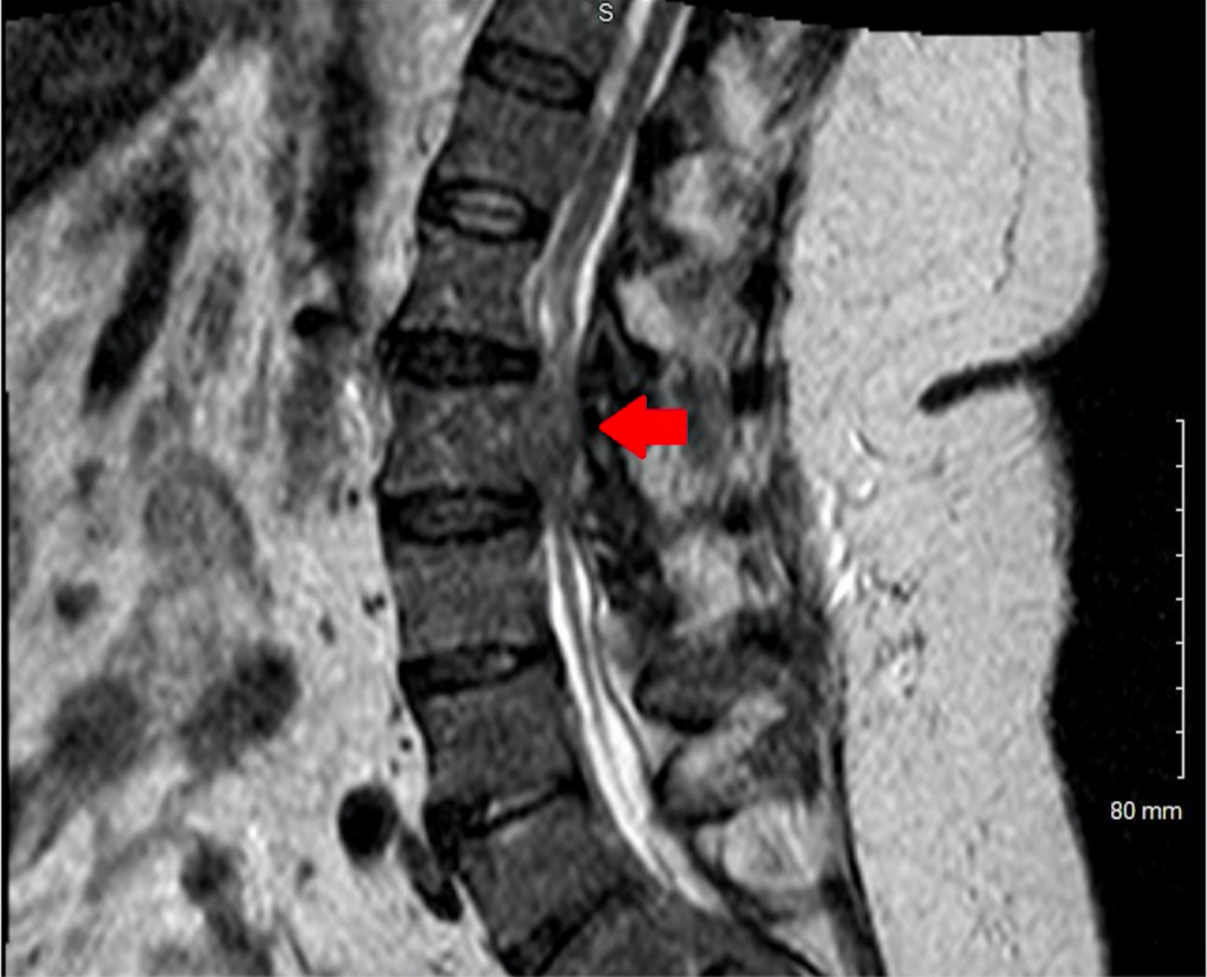
MRI lumbar spine - lesion (red arrow) at L2 level causing compression of cauda equina.

She then underwent MRI of the thoracic and lumbar spine with and without IV contrast, which showed extensive epidural and possibly subdural enhancement at multiple levels of canal. A lesion within the canal at L2 level was causing severe compression of the cauda equina just below the level of conus and extending into the right foramen at L2-3. These findings were highly suggestive of a neoplastic process (Figure 1).

The patient was admitted to the hospital with the diagnosis of cauda equina syndrome. Neurosurgery was consulted, and she went to operating room emergently and underwent L1-3 laminectomy and de-bulking of the mass. The tissue was sent to pathology and cultures. While pathology of the mass was pending, the patient had a urinary catheter placed for her urinary retention. She also had acute kidney injury at the time of presentation which was thought to be multifactorial. Her creatinine level went up over the next two days, but later on normalized with IV fluids.

Biopsy of the tissue from the spine revealed BL with cytogenetics showing 8;14 translocation. Furthermore, cultures from the same tissue grew methicillin resistant staphylococcus aureus (MRSA).

Oncology was consulted at this time and the patient underwent further imaging to evaluate the extent of cancer burden with CT scan of neck, chest, abdomen, and pelvis. She was noted to have involvement of terminal ileum. She also underwent bone marrow biopsy which showed high grade B-cell lymphoma. She was started on rasburicase for elevated uric acid and prevention of tumor lysis syndrome. Oncology team recommended urgent initiation for treatment of BL. Lumbar puncture was performed for intrathecal administration of methotrexate (MTX), but needle could not be placed in ideal position after multiple attempts. However, small amount of cerebrospinal fluid (CSF) was collected, which eventually was positive for high grade lymphoma. Further results of CSF analysis are shown in Table 2.

**Table 2:** CSF analysis. CSF, cerebrospinal fluid

Test name	Results	Reference range
Glucose	36 (L)	50-75 mg/dL
Protein, total	388 (H)	15-45 mg/dL
Color	Yellow	N/A
CSF character	Slightly hazy	N/A
Total nucleated cells	15 (H)	0-5/uL
Red blood cells	9,875	0-10/uL
Polymorphonuclear cells	32	N/A
Mononuclear cells	20	N/A

Infectious Diseases was consulted given the presence of MRSA in tissue cultures. Even though, she did not have any signs of infection negative blood cultures, she was started on IV vancomycin given her immunocompromised state due to lymphoma currently and chemotherapy later.

Given the complexity of the patient's clinical status and potential complications of the treatment of BL, the patient was transferred to a quaternary hospital for further management and treatment.

After transfer to the quaternary hospital, the patient received five treatments of radiation therapy to spinal lesions and one cycle of R-CHOP (Rituximab, Cyclophosphamide, Hydroxydaunomycin, Oncovin, Prednisolone) chemotherapy. She received three doses of MTX and two doses of cytarabine intrathecally in regular intervals after placement of an Ommaya reservoir. She also received one cycle of R-EPOCH (Rituximab, Etoposide, Prednisolone, Oncovin, Cyclophosphamide, Hydroxydaunorubicin) therapy. She continued to receive rasburicase with improvement in uric acid levels. IV vancomycin was continued for a total of two weeks. Furthermore, she received appropriate bacterial, viral, and fungal prophylaxis and bone marrow stimulating agents due to chemotherapy.

After one month of prolonged hospitalization, she was then discharged to a rehabilitation center with scheduled weekly alternate intrathecal MTX and cytarabine. She continued to stay in a rehabilitation center and received multiple rounds of intrathecal MTX and cytarabine as well as more cycles of R-EPOCH chemotherapy. 

Five months later she was admitted to the hospital due to sepsis secondary to a urinary tract infection and severe encephalopathy. At that time she was found to have new renal masses consistent with relapse of the lymphoma. During that admission, she was given comfort care and passed away.

## Discussion

Numb chin syndrome most frequently occurs due to odontogenic causes [5]. These causes range from local trauma, fractures, cysts, or osteomyelitis of the mandible and dental abscesses. It had also been seen to be present in systemic infections and inflammatory diseases such as HIV, syphilis, Lyme disease, sarcoidosis, multiple sclerosis, systemic lupus erythematosus, scleroderma, etc. [6]. However, the significance of this syndrome is due to its association with malignancies which was first reported by Calverley and Mohnac, who reported five cases in which NCS was the initial presentation for an underlying metastatic malignant disease [7]. NCS has been associated with hematological as well as with solid malignancies. It is most commonly associated with metastatic breast cancer and lymphoma, followed by prostate cancer, lung cancer, and leukemia [8]. In a retrospective study of patients with NCS, breast cancer comprised 64% of the primary tumors, and lymphoproliferative neoplasms comprised 14% [9].

Numb chin syndrome related to malignancy can either be unilateral or bilateral [9]. But more cases of bilateral NCS are associated with hematological malignancies than with solid malignancies. BL in particular is associated with bilateral NCS [8]. In the present case, the patient also had bilateral numbness of the chin and after the diagnosis of BL it was concluded that NCS was related to it. In addition to the timing of onset of numbness in our case, bilateral involvement also excluded the possibility of its relation to recent dental procedure and associated left-sided local anesthesia. Furthermore, NCS precedes a diagnosis of malignancy in up to 47% of cases [10] and is usually the first manifestation in hematological malignancies in general and BL in particular [8]. In our case, even though the patient presented with clinical manifestation of cauda equine syndrome related to mass compression of spinal cord, chronologically the numbness of chin was the first symptom.

Majority of cases arising from neoplastic etiologies can be explained by either local compression or neoplastic infiltration of the mental or inferior alveolar nerves [11]. However, pathophysiology of NCS in relation to distant malignancies such as lymphoma has not been completely understood. It is plausible that in cases of distant neoplasms, it may be related to hematogenous, lymphatogenous, or even leptomeningeal spread. In a review of 36 patients with NCS, Lossos and Siegal reported 50% of the patients had mandibular metastases, 14% base-of-skull bone lesions, and 22% leptomeningeal seeding. The patients with leptomeningeal seeding had malignant cells in the CSF and otherwise normal evaluations [9]. Of note, Raaphorst and Vanneste reported a case of numb cheek syndrome as the anti-Hu paraneoplastic neuronopathy [12], hinting that NCS may also carry similar paraneoplastic or autoimmune pathogenesis. Moreover in some cases the cause may not be found at all as in the review by Lossos and Siegal, where the cause was unknown in 11% of patients [9]. In our patient, NCS most likely happened as a result of leptomeningeal spread given the presence of neoplastic cells in the CSF and evidence of neoplastic process on multiple epidural and subdural levels on MRI.

The presence of NCS in association with the malignancy carries a poor prognosis. Its occurrence in patients with malignancy is indicative of metastasis, with a reported overall mortality of 79% and a weighted mean survival of approximately seven months [13]. Our patient passed away approximately six months after the diagnosis of BL. It is crucial to view NCS as a warning sign that is indicative of a poor survival outcome when it is present in patients with underlying malignant diseases [14]. At the same time, NCS in the absence of any identifiable cause should raise suspicion for underlying malignancy and brain imaging should be pursued to look for metastasis. CT or an MRI of the brain may show metastatic brain lesions or even leptomeningeal involvement; however, MRI is considered superior to CT scan while evaluating the etiology of NCS [1].

## Conclusions

Numb chin syndrome can be the first sign of underlying malignancy or recurrence of a malignancy. Its presence in the setting of an underlying malignancy indicates very poor prognosis as in this case. Clinicians should have a very high index of suspicion to diagnose NCS. Absence of any identifiable causes should trigger thorough workup for occult malignancy as NCS can be the initial symptom of malignancy.
